# Letter to the editor: results from a Web‐based survey to identify dynapenia screening tools and risk factors

**DOI:** 10.1002/jcsm.12128

**Published:** 2016-08-23

**Authors:** Todd M. Manini, Brian C. Clark

**Affiliations:** ^1^ Institute on Aging, Department of Aging and Geriatric ResearchUniversity of FloridaGainesvilleFLUSA; ^2^ Ohio Musculoskeletal and Neurological Institute (OMNI), Department of Biomedical SciencesOhio UniversityAthensOHUSA

## Conflict of interest

Todd M Manini and Brian C Clark declare they have no conflict of interest.

There is increased focus on using muscle strength as an indicator of clinically relevant muscle weakness.^1^, ^2^ However, there is little information about how to efficiently screen patients who might have a risk of clinically significant muscle weakness. We identified potential respondents by entering combinations of the following search terms: geriatrics, sarcopenia, dynapenia, physical function, aging, muscle strength, muscle power, and/or physical disability in Medline. We presumed that the authors of these papers would be able to provide insight on screening for muscle weakness. We then located email contact information for each author. Drs Clark and Manini personally invited 212 authors to complete a brief Web‐based survey that asked questions concerning screening for risk factors related to age‐related muscle weakness that we referred to as *dynapenia*.^3^ The respondents were asked to provide answers to an anonymous Web‐based poll that remains active (http://www.dynapenia.blogspot.com/).^4^ The poll asked the following questions: (i) ‘Is grip strength an appropriate screening tool?’, (ii) ‘Is a knee extension strength test appropriate for defining dynapenia?’, (iii) ‘Should age be considered an absolute indication for screening for dynapenia?’, and (iv) ‘Please pick the 5 most predictive risk factors for dynapenia’. Twenty‐four people responded to the survey, and there were 127 responses for risk factors of dynapenia.

We found that 50% (*n* = 12) of the respondents considered grip strength an appropriate screening tool, whereas 16% considered it unnecessary, and 16% considered it to be a poor predictive factor. Similarly, 57% of the respondents considered knee extension strength appropriate to define dynapenia, while 28% instead recommended walking speed, 9% suggested multi‐joint testing, and 4% recommended muscle power, respectively, as better tests to define dynapenia. Moreover, 54% of the respondents did not consider age as an absolute indicator of screening for dynapenia, while 18% believed it to be for age >55 years old, 13% considered that it should be for people aged >65 years old, and 9% considered that it should be for people aged >75 years old.

**Figure 1:**
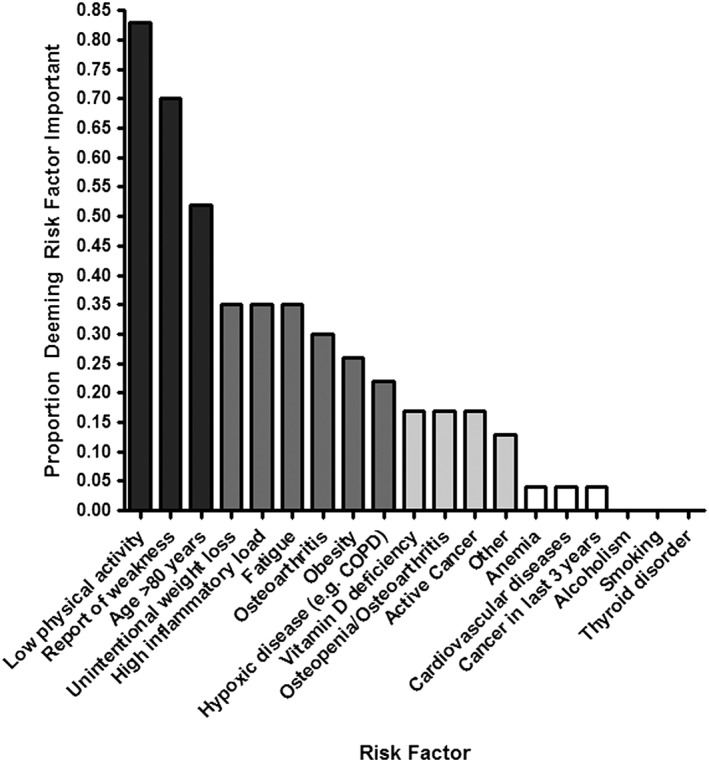
Proportion responding to the five most predictive risk factors for dynapenia.

There were 127 responses to the top five most predictive risk factors for dynapenia (Figure 1). In order of highest percentage positive response, the respondents chose low physical activity, report of weakness, age >80 years, unintentional weight loss, and high inflammatory load as the top five most predictive risk factors (*n* = 127). Sequential Chi‐square tests of proportions identified that the responses were clustered into four major groups that are identified by colour in Figure 1. Low physical activity, a report of muscle weakness, and being >80 years of age clustered into the most prevalent responses. Unintentional weight loss, inflammation, fatigue, osteoarthritis, obesity, and hypoxic disease formed a second cluster. Vitamin D deficiency, osteoporosis, active cancer, and other conditions formed a third cluster. Selections for anaemia, cardiovascular disease, cancer in last 3 years, alcoholism, smoking, and thyroid conditions were the least frequently chosen.

This Web‐based survey provides information about the opinions of a small, but select group of respondents publishing in the field of geriatrics, gerontology, ageing, muscle, and physical function. Recently, grip strength—because of ease of use, predictive capability, and robust use across many studies—has been identified as a potential screening tool to label older adults with clinically significant muscle weakness.^5^ Approximately half of the respondents felt that grip strength was an appropriate tool for screening. These opinions might be different today, considering that the survey was completed prior to latest knowledge provided by the FNIH Sarcopenia Project.^6^ We might expect this rate to increase as more knowledge about the screening capabilities of measuring grip strength is put forth in the literature.

While this Web survey asked the opinions of the respondents who publish in the field, the results cautioned to be directly used without large representation from the clinical community. We acknowledge that because this survey was available to the public, individuals without specific expertise on sarcopenia/dynapenia could have provided responses. Identifying older adults at risk of physical disability using various sarcopenia and clinically significant weakness definitions is an emerging area of research. As such, these results may provide a guide for practitioners to understand the opinions of respondents who are likely to be experts in the field. The aim is to help initiate a brief set of questions that can be efficiently administered by practitioners.

### Conclusions

Grip strength and knee extension strength were considered appropriate tools to screen dynapenia by a majority of respondents. The respondents chose low physical activity, reported weakness, and age >80 years as being the most important risk factors for dynapenia. The results are expected to help develop a simple question‐based screening approach to gauge a patient's risk of dynapenia.

### Acknowledgements

The authors have complied with the guidelines of ethical authorship and publishing as stated in the Journal of Cachexia, Sarcopenia and Muscle: update 2015).^7^ This work was supported by the following grants from the National Institutes of Health's National Institute on Aging: R01 AG042525, R01 AG044424, and the University of Florida, Claude D. Pepper Center (P30 AG028740). This work was considered non‐human research, and therefore, was exempt from ethical standards laid down in the 1964 Declaration of Helsinki and its later amendments. This work was sponsored by the National Institutes of Health. The sponsors had no role in the concept, design, methods, and analysis or preparation of paper.
